# Cytomegalovirus Infection and Clinical Outcomes in Hospitalized Chimeric Antigen Receptor T-Cell Therapy Recipients

**DOI:** 10.7759/cureus.101121

**Published:** 2026-01-08

**Authors:** Muhammad Atif Khan, Muhammad Shafiq, Mehran Ali Khan, Faiza Humayun Khan, Joseph P McGuirk, Hayrettin Okut, Edward Ellerbeck, Nausheen Ahmed

**Affiliations:** 1 Internal Medicine, University of Kansas Medical Center, Kansas City, USA; 2 Medicine, Nowshera Medical College, Nowshera, PAK; 3 Internal Medicine, Montefiore St. Luke's Cornwall, Newburgh, USA; 4 Hematologic Malignancies and Cellular Therapeutics, University of Kansas Medical Center, Kansas City, USA; 5 Preventive Medicine, University of Kansas School of Medicine-Wichita, Wichita, USA; 6 Population Health, University of Kansas Medical Center, Kansas City, USA

**Keywords:** chimeric antigen receptor (car) t-cell, cytomegalovirus (cmv), incidence, length of hospital stay (los), mortality

## Abstract

Background: Chimeric antigen receptor T (CAR-T) cell therapy has revolutionized the management of hematologic malignancies but is associated with significant toxicities, including opportunistic infections such as cytomegalovirus (CMV). Limited evidence exists regarding the clinical impact of CMV in CAR-T recipients. This study evaluated outcomes associated with CMV infection in this population.

Methods: A retrospective cohort study was conducted using the 2021 Healthcare Cost and Utilization Project-National Readmissions Database (HCUP-NRD). Adult patients hospitalized for CAR-T therapy for multiple myeloma, non-Hodgkin lymphoma, or acute leukemia were included. Propensity score matching was applied to balance baseline characteristics, and weighted analyses were conducted in R software (R Foundation for Statistical Computing, Vienna, Austria) to compare outcomes between CMV-positive and CMV-negative groups.

Results: Among 1,806 hospitalizations, CMV infection was identified in 2.2% of patients during the index admission. In the matched cohort, in-hospital mortality in the CMV group compared with non-CMV patients (10.3% vs. 2.6%; risk ratio (RR) 3.9, 95% CI: 0.46-33.3; p = 0.36). CMV infection was associated with significantly longer length of stay (41.5 vs. 15.9 days; adjusted ratio 1.67, 95% CI: 1.27-2.19; p < 0.01) and increased risk of encephalopathy (RR 13.21, 95% CI: 1.66-105.2; p = 0.015). Other complications, including cytokine release syndrome, tumor lysis syndrome, acute kidney injury, and transfusion requirements, did not differ significantly. At three months post-CAR-T, the cumulative incidence of patients hospitalized with CMV infection was 4.2%. Three-month mortality was significantly higher in CMV patients compared with non-CMV patients (15.8% vs. 2.5%; RR 6.32, 95% CI: 1.46-27.3; p = 0.004).

Conclusion: CMV infection in CAR-T recipients is associated with increased in-hospital mortality, extended hospital stays, and a higher risk of encephalopathy. These findings underscore the importance of vigilant monitoring and early management of CMV in this high-risk population.

## Introduction

Chimeric antigen receptor T (CAR-T) cell therapy represents a groundbreaking innovation in the treatment of hematologic malignancies, including acute B-cell lymphoblastic leukemia (B-ALL) and non-Hodgkin lymphoma (NHL), specifically large B-cell lymphoma (LBCL) and mantle cell lymphoma (MCL), as well as multiple myeloma (MM) [1-11]. This pioneering therapy has demonstrated promising complete remission rates of 80%, 52%, 67%, and 67% for the respective hematologic malignancies mentioned [3,11-13]. However, despite encouraging responses, there are significant toxicities, notably cytokine release syndrome (CRS) and immune effector cell-associated neurotoxicity syndrome (ICANS) [14,15]. To manage these toxicities, patients are treated with steroids and cytokine inhibitors. These medications mitigate side effects, further affecting immune reconstitution. As a result, both CAR-T cells and supportive medications used to manage their toxicities increase patients' susceptibility to infections. This leads to a high infection rate of 23-42%, particularly within the first 30 days following CAR-T cell therapy [16,17]. Bacterial infections are the most common, occurring in 12-17% of cases, followed by viral (5-9%) and fungal (3% to 8%) [16-20]. These infections are significant contributors to non-relapse mortality (NRM). The NRM rate associated with CAR-T cell therapy is approximately 6.8%, with infections accounting for a significant proportion, contributing to approximately 50.9% of these cases [21].

Cytomegalovirus (CMV) is a well-recognized pathogen that can be reactivated in immunocompromised individuals, including recipients of hematopoietic stem cells (HSCT) and solid organ transplants due to impaired adaptive immunity. Studies indicate that CMV reactivation occurs in 23-69% of allogeneic HSCT recipients [22-24]. CMV infection is associated with increased morbidity and mortality in post-transplant patients [25-28]. Given the poor outcomes associated with CMV infections in immunocompromised populations, there is a growing interest in investigating the impact of CMV infection on patients following CAR-T therapy.

CD8-positive cytotoxic T cells play a crucial role in controlling primary and secondary viral infections, including CMV [29]. The lymphocyte depletion therapy used in CAR-T therapy, while creating a favorable immune environment for CAR-T cell proliferation and response, also increases the risk of CMV reactivation due to the absence of CD8+ cell-mediated surveillance [30]. The incidence of CMV reactivation in CAR-T cell therapy patients is comparable to that in other immunocompromised conditions, ranging from 22% to 33% [31-34]. Notably, CMV reactivation is associated with higher NRM in these patients, with a rate of 57% compared with 23% in those without CMV reactivation [33]. This finding highlights the critical impact of CMV reactivation on the prognosis of CAR-T cell therapy recipients. Some risk factors that have been found to be associated with CMV infection in patients after CAR-T cell therapy include lactate dehydrogenase levels at the time of CAR-T cell therapy, severity of ICANS or CRS, and treatment of CRS or ICANS with steroids, tocilizumab, or anakinra [32,33].

Although CMV reactivation has been shown to be associated with increased NRM and prolonged hospitalization, its full impact on CAR-T recipients remains underexplored. The current literature, primarily based on single-center studies, lacks comprehensive and generalizable data regarding clinical outcomes of CMV infection in this patient population, particularly concerning mortality rates and associations with other treatment-related complications. We utilized the National Readmission Database (NRD) database, which provides a broad, representative sample size encompassing multiple institutions, offering a more comprehensive analysis [35]. This study aimed to fill these gaps by evaluating the outcomes of CMV infection in hospitalized CAR-T therapy recipients, offering valuable insights for improving patient management and clinical decision-making.

## Materials and methods

Data source

This study used the 2021 NRD, a part of the Healthcare Cost and Utilization Project (HCUP) developed by the Agency for Healthcare Research and Quality (AHRQ). The NRD is a publicly available, de-identified, large all-payer inpatient care database that provides a nationally representative sample of U.S. hospitalizations. It contains discharge-level data from 31 geographically diverse states, covering approximately 62% of the U.S. population and 61% of all hospital discharges. Each hospitalization is linked with patient-level identifiers that allow tracking of readmissions across hospitals within the same state during a calendar year. The NRD includes demographic variables, diagnostic and procedure codes, comorbidity information, length of stay (LOS), discharge disposition, and inpatient mortality. Outpatient visits are excluded.

The NRD was selected for this study because it captures nationally representative inpatient outcomes, including LOS and in-hospital mortality, allows three-month follow-up for readmissions within the same year, and supports propensity score matching (PSM), a rigorous statistical method employed to minimize the effects of confounding and selection bias in our observational analysis. As the data within the NRD are de-identified and publicly available under a data use agreement, institutional review board (IRB) approval was not required.

Study population and selection criteria

Inclusion Criteria

Inclusion criteria were adult patients (≥18 years) admitted to U.S. hospitals in 2021; receipt of FDA-approved CAR-T cell therapy identified using International Classification of Diseases, 10th Revision, Procedure Coding System (ICD-10-PCS) procedure codes XW033C3, XW043C3, XW033C7, and XW043C7; and indications included MM, NHL, or acute leukemia (AL).

Exclusion Criteria

Exclusion criteria included patients with a documented CMV infection prior to CAR-T infusion (to ensure inclusion of only new infections after therapy) and patients discharged between October and December 2021, to allow a full three-month follow-up period.

Group definitions

The index hospitalization was defined as the admission during which CAR-T infusion occurred. Patients were divided into two groups.

The CMV group included patients with an ICD-10-CM code for CMV infection (B25 or B271) during index admission. These broad codes capture unspecified CMV infection and organ-specific disease (lungs, liver, intestine, retina, and others). Specific codes for CMV viremia or colitis were not available.

The non-CMV group included patients without CMV diagnostic codes during the study period.

Because specific CAR-T-related ICD-10 codes were introduced only in October 2021, a general CAR-T procedure code was used to ensure complete year-long capture. This approach included all FDA-approved CAR-T products available in 2021: axicabtagene ciloleucel, tisagenlecleucel, lisocabtagene maraleucel, brexucabtagene autoleucel, and idecabtagene vicleucel.

Exploratory analysis population

The exploratory analysis used the same CAR-T cohort but followed patients for three months after the index hospitalization to capture rehospitalizations with CMV infection and subsequent in-hospital mortality. For this analysis, the CMV group included patients who had a CMV ICD-10 code either during the index admission or within any rehospitalization in the three-month period, while the non-CMV group included those without CMV codes during the same follow-up window.

Outcomes

Primary Outcome

The primary outcome was inpatient mortality during the index hospitalization in patients with CMV infection compared with those without CMV infection.

Secondary Outcomes

Secondary outcomes were LOS on index hospitalization and CAR-T-related complications including CRS, encephalopathy (used as a surrogate for ICANS due to absence of specific ICD-10 code in 2021), tumor lysis syndrome (TLS), acute kidney injury (AKI), hemophagocytic lymphohistiocytosis (HLH), transaminitis, red blood cell (RBC) transfusion, and platelet transfusion.

Exploratory Outcomes (Three-Month Follow-Up)

Exploratory outcomes were incidence of CMV infection within three months post-CAR-T and mortality during the index admission plus within three months post-discharge.

Statistical analysis

Data extraction was performed using the “dplyr” package in R version 4.3.3 (R Foundation for Statistical Computing, Vienna, Austria). Patient demographics (age, sex), clinical variables, LOS, and in-hospital mortality were directly obtained from NRD.

Weighted survey analysis was applied to account for the complex NRD sampling design. PSM was used to minimize baseline differences between CMV and non-CMV groups. Matching was performed in a 1:1 ratio without replacement using the optimal method, with a caliper width of 0.1. Covariates in the exposure model included age, sex, Charlson Comorbidity Index (CCI), and baseline comorbidities such as congestive heart failure (CHF), coronary artery disease (CAD), cerebrovascular disease (CVD), pulmonary hypertension, hypertension, hyperlipidemia, diabetes mellitus (DM), chronic kidney disease (CKD), asthma, and chronic obstructive pulmonary disease (COPD). Survey design variables (discharge weight, NRD stratum, NRD hospital ID) were also incorporated.

For Matched Cohorts

Mortality: Treated as a categorical variable (death vs no death), was compared between groups using Fisher’s exact test with survey weights.

LOS: Treated as continuous variable, was analyzed using a survey-weighted generalized linear model (GLM) with a Gamma distribution and log link function. Effect estimates are reported as adjusted mean ratio with 95% confidence Intervals (CIs) and p-values.

Major complications: Treated as binary variables, the incidence of complications (CRS, encephalopathy, TLS, AKI, transaminitis, and transfusions) was assessed using survey-weighted binomial regression.

Subset and sensitivity analyses were not performed due to limited sample size, which could compromise validity.

## Results

**Figure 1:**
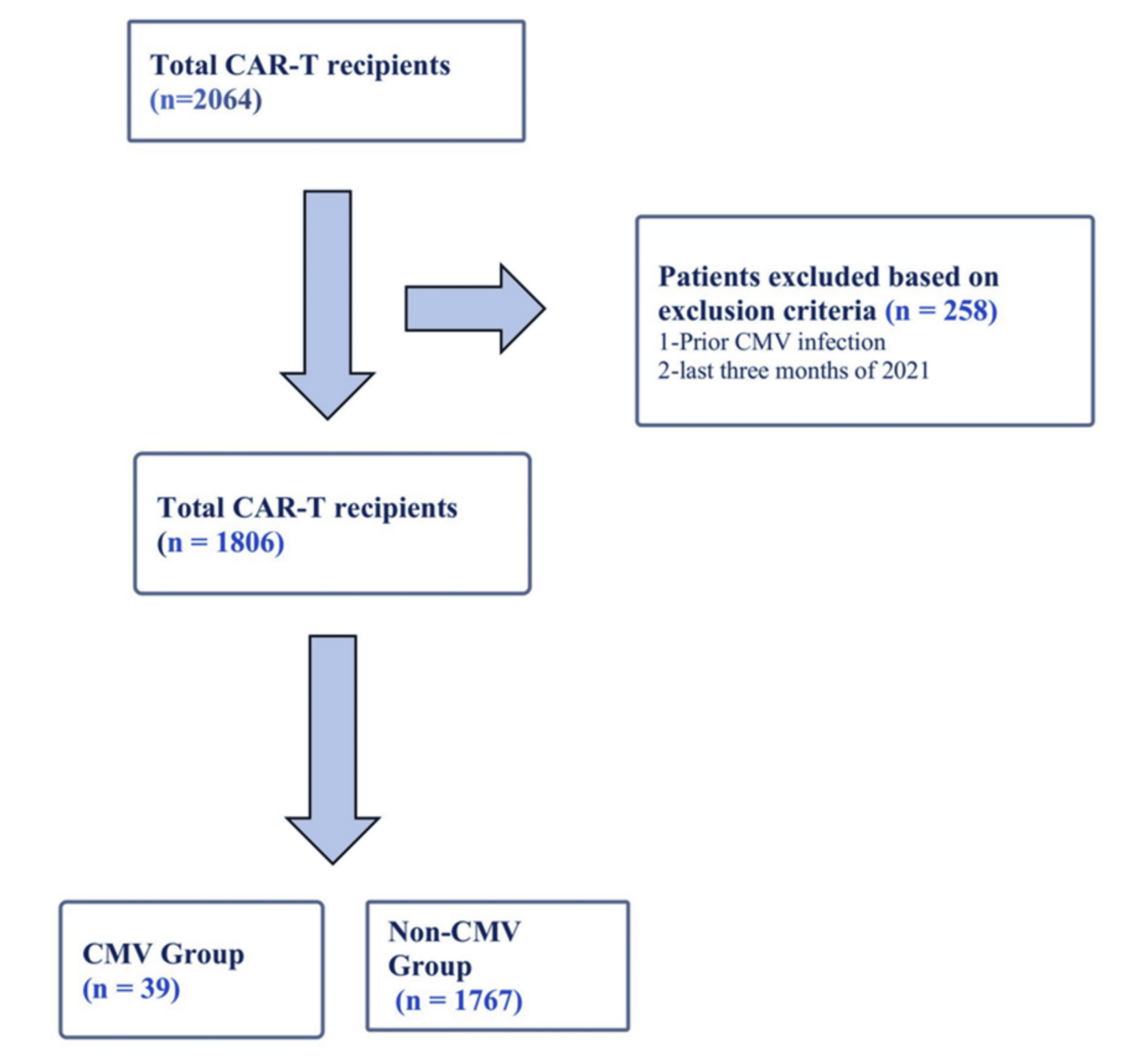
Patient Selection and Study Cohort CAR-T: chimeric antigen receptor T cell, CMV: cytomegalovirus

The estimated total hospitalizations in 2021 in which adult patients received CAR-T cell therapy for either MM, NHL, or AL were 2064, of which 258 hospitalizations were excluded based on the exclusion criteria. The estimated final weighted cohort represented 1806 hospitalizations (Figure 1). The mean age of the patients receiving CAR-T therapy was 61.9 years, with males comprising 64.4% of the sample. The most common comorbidity was HTN (53.4%), followed by HLD (33.9%) and DM (19.6%) (Table 1). Approximately half of the patient population had a CCI of 2 (48.7%), followed by 3 (21.9%), suggesting a relatively lower burden of chronic diseases. The majority of patients were treated for NHL (73.7%), followed by MM (21.6%) and AL (4.7%) (Table 1).

**Table 1:** Baseline characteristics of CAR-T therapy recipients. CAR-T: chimeric antigen receptor T cell, CHF: congestive heart failure, HTN: hypertension, CAD: coronary artery disease, CVD: cerebrovascular disease, HLD: hyperlipidemia, DM: diabetes mellitus, CKD: chronic kidney disease, COPD: chronic obstructive pulmonary disease

Variable – n (%)	Unweighted	Weighted estimate
All admissions	966	1806
Age, years (mean)	61.7	61.9
Male sex	617 (63.9)	1163 (64.4)
Underlying disorder		
Acute leukemia	49 (5.1)	85 (4.7)
Multiple myeloma	208 (21.5)	390 (21.6)
Non-Hodgkin lymphoma	709 (73.4)	1331 (73.7)
Congestive heart failure	67 (6.9)	142 (7.9)
Pulmonary hypertension	25 (2.6)	53 (2.9)
Coronary artery disease	96 (9.9)	172 (9.5)
Cerebrovascular disease	81 (8.4)	147 (8.2)
Hypertension	505 (52.3)	965 (53.4)
Hyperlipidemia	322 (33.3)	613 (33.9)
Diabetes mellitus	178 (18.4)	353 (19.6)
Chronic kidney disease	70 (7.3)	141 (7.8)
COPD / emphysema	47 (4.9)	99 (5.5)
Asthma	58 (6.0)	111 (6.1)

CMV infection occurred in 2.2% of patients during index hospitalization (n = 39), while the remaining patients comprised the non-CMV group (n = 1767). Following PSM, no significant differences were observed in baseline characteristics between the groups (Table 2). The matched cohort included 77 hospitalizations for CAR-T, with 39 CMV-positive and 38 CMV-negative patients.

**Table 2:** Weighted Baseline Characteristics of the CMV and Non-CMV Groups CMV: cytomegalovirus, PSM: propensity score matching, CHF: congestive heart failure, HTN: hypertension, CAD: coronary artery disease, CVD: cerebrovascular disease, HLD: hyperlipidemia, DM: diabetes mellitus, CKD: chronic kidney disease, ESRD: end-stage renal disease, COPD: chronic obstructive pulmonary disease

Variables	Before PSM Matching			After PSM Matching		
	CMV positive (n = 39)	CMV negative (n=1767)	p-value	CMV positive (n = 39)	CMV negative (n = 38)	p-value
AGE =mean (SD)	63.62 (8.43)	61.89 (12.49)	0.344	63.62 (8.43)	64.96 (9.97)	0.63
Male Sex, n(%)	33 (84.6)	1130 (64.0)	0.023	33 (84.6)	31 (81.6)	0.77
CHF, n(%)	6 (15.4)	136 (7.7)	0.31	6 (15.4)	2 (5.3)	0.24
Pulmonary HTN, n(%)	0( 0.0)	53 (3.0)	0.55	0 ( 0.0)	0 (0.0)	na
CAD, n(%)	5(12.8)	167(9.5)	0.64	5(12.8)	10(26.3)	0.28
HTN, n(%)	34(87.2)	931(52.7)	< 0.001	34(87.2)	33(86.8)	0.99
HLD, n(%)	16(41.0)	597(33.8)	0.39	16(41.0)	17(44.7)	0.9
CVD, n(%)	6(15.4)	141(8.0)	0.12	6 (15.4)	9( 23.7)	0.4
DM, n(%)	15(38.5)	339(19.2)	0.005	15(38.5)	15(39.5)	0.91
CKD, n(%)	6(15.4)	135(7.6)	0.31	6(15.4)	4(10.5)	0.64
ESRD, n(%)	5(12.8)	3(0.2)	< 0.001	5 (12.8)	0 (0.0)	0.22
COPD/Emphysema, n(%)	2 (5.1)	97 (5.5)	0.94	2(5.1)	13(34.0)	0.04
Asthma, n(%)	0(0.0)	111(6.3)	0.4	0( 0.0)	0 ( 0.0)	na

Primary outcome

Among the matched patients during the index hospitalization, four patients (10.3%) died in the CMV group. One patient (2.6%) in the non-CMV group died. This difference was not statistically significant (risk ratio (RR) of 3.9, 95% CI: 0.46 - 33.3, p =0.36) (Table 3).

**Table 3:** CMV Index Hospitalization Outcomes *Descriptive only; primary inference based on generalized linear model (GLM) ratio due to skewed distribution. CMV: cytomegalovirus

Outcome	CMV Group	Non-CMV Group	Risk Ratio (RR) / Adjusted Ratio
Incidence of CMV infection (Index hospitalization)	2.2%	N/A	N/A
Mortality (Index hospitalization)	10.3%	2.6%	RR = 3.9 (95% CI: 0.46 - 33.3, p=0.36)
Length of Stay (mean)	41.5 days *	15.9 days *	Adjusted ratio = 1.67 (95% CI: 1.27 – 2.19, p<0.01)

Secondary outcomes

The estimated mean LOS during the index admission was 41.5 days for patients diagnosed with CMV, when compared to the estimated mean LOS of 15.9 days for patients without a diagnosis of CMV (Table 3). Because of the skewed distribution of LOS, a survey-weighted generalized linear modeling with a gamma distribution and log link was used for inference. After adjusting for CRS, encephalopathy, TLS, HLH, AKI, transaminitis, RBC transfusion, and platelet transfusion, CMV infection remained independently associated with a significantly longer LOS. Patients in the CMV group had 1.67 times the LOS compared to non-CMV patients (95% CI: 1.27-2.19, p < 0.001) (Table 3).

Patients in the CMV group had higher rates of encephalopathy, with a RR of 13.21 (95% CI: 1.66-105.2, p=0.015), although no encephalopathy-related deaths occurred during the index admission (Table 4). Rates of other complications, including TLS, AKI, RBS, and platelet transfusion, were also higher in the CMV group compared to the non-CMV group; however, these differences did not reach statistical significance (Table 4).

**Table 4:** Risk ratio for complications in CMV infected CAR-T therapy recipients. CAR-T: chimeric antigen receptor T cell, CRS: cytokine release syndrome, TLS: tumor lysis syndrome, AKI: acute kidney injury, RBC: red blood cell, CMV: cytomegalovirus

Complications	CMV (n=39)	Non CMV (n=38)	Risk ratio (RR)	95 % confidence interval	p-value
CRS	30(76.9%)	30(78.9%)	0.97	0.65 – 1.44	0.88
Encephalopathy	16(41%)	1(2.6%)	13.21	1.66 – 105.2	0.015
TLS	3(7.7%)	2(5.3%)	1.31	0.11 – 15.15	0.22
AKI	9(23.1%)	1(2.6%)	6.17	0.64 – 59.86	0.12
Transaminitis	2(5.1%)	2(5.3%)	0.98	0.44 – 2.14	0.95
RBC Transfusion	7(17.9%)	5(13.2%)	1.41	0.35 – 5.76	0.63
Platelet Transfusion	6(15.4%)	3(7.9%)	2.35	0.62 – 8.94	0.21

Exploratory outcomes

In the exploratory three-month follow-up analysis, the population included all CAR-T recipients with adequate follow-up time to capture rehospitalizations after discharge. Over this extended period, the incidence of CMV infection increased to 4.2%, representing 76 patients, reflecting those who developed CMV either during the index hospitalization or during a rehospitalization within three months (Table 5).

**Table 5:** CMV Three-Month Follow-up Outcomes CMV: cytomegalovirus

Outcome	CMV Group	Non-CMV Group	Risk Ratio (RR)
Incidence of CMV infection (three-month follow-up)	4.2%	N/A	N/A
Mortality (three-month follow-up)	15.8%	2.5%	RR = 6.32 (95% CI: 1.46 - 27.3, p=0.004)

After PSM, the three-month follow-up cohort consisted of 156 total patients, with 76 patients in the CMV group and 80 patients in the non-CMV group. During this period, 12 patients (15.8%) in the CMV group died compared with two patients (2.5%) in the non-CMV group. This corresponds to a significantly higher risk of mortality among patients who developed CMV, with a risk ratio of 6.32 (95% CI, 1.46-27.30; p = 0.004) (Table 5).

## Discussion

This study underscores the outcomes of CAR-T therapy in patients with CMV infection and their influence on associated complications. In this cohort, the predominant underlying condition was NHL (73.7%), followed by MM (21.6%) and AL (4.7%), consistent with the general incidence trends reported by the Surveillance, Epidemiology, and End Results (SEER) program [36]. The high proportion of NHL reflects its prevalence as the primary indication for CAR-T therapy and provides a framework for interpreting the impact of CMV infection across different malignancies, offering insights into potential disease-specific risks and outcomes.

The incidence of CMV infection among hospitalized CAR-T recipients in our study was 4.2% within three months post-therapy, including 2.2% identified during the index admission. We know from previous reports that the overall incidence of CMV is around 10%, as reported by Khawaja et al. [32]. This highlights that most cases are detected through surveillance, managed preemptively on an outpatient basis, and therefore not represented in this study. Moreover, this difference may be attributed to the shorter follow-up duration of three months in this study compared to the 12-month follow-up in theirs [32]. Among the hospitalized patients, we noted that the mortality rate was significantly higher in the CMV-infected group, with 12 deaths (15.8%) during the three-month follow-up, compared to just two in the non-CMV group (risk ratio of 6.32, p=0.004). A clinically meaningful difference was also observed at index admission, with mortality of 10.3% in CMV patients versus 2.6% in the non-CMV group. No deaths were observed in CMV patients with encephalopathy. This finding suggests that CMV infection is significantly associated with higher mortality in CAR-T recipients which also aligns with the higher mortality rate reported by Lin et al. [33]. Additionally, the extended LOS for CMV-infected patients, with an adjusted ratio of 1.67 (p= <0.01) compared to non-CMV patients, suggests that CMV infection may have a substantial impact on the clinical course of CAR-T recipients. CMV infection is commonly treated with oral ganciclovir formulations, which help facilitate a shorter hospital stay [37]. However, the emergence of resistant CMV strains presents a new challenge, with limited therapeutic options such as foscarnet and cidofovir often resulting in prolonged hospitalizations [38].

The majority of the patients were male (64.4%), with an average age of 61.9 years. This pattern of age and gender distribution has remained consistent across most studies reflecting the epidemiology of the underlying malignancies commonly treated with CAR-T [31-33,39]. Comorbid conditions such as HTN (53.4%), HLD (33.9%), and DM (19.6%) were prevalent, with the majority of patients having a CCI of 2 or 3, reflecting the typical comorbidity burden in CAR-T recipients, similar across the CMV and non-CMV groups. These comorbidities are crucial to consider, as they may contribute to adverse outcomes, including infections such as CMV reactivation, and influence the overall clinical course of CAR-T therapy. This could also impact treatment decisions for CMV, given the established association of foscarnet with renal dysfunction and ganciclovir with neutropenia [40,41]. We found no baseline characteristics linked to adverse outcomes in CMV infection, except for encephalopathy/ICANS.

Analysis of the relationship between CMV infection and other complications indicated a higher incidence of encephalopathy among patients with CMV infection, possibly driven by increased steroid use, which may contribute to a higher risk of CMV infection. This aligns with the higher incidence of ICANS reported by Khawaja et al. [32]. However, other common complications, such as CRS, AKI, and transaminitis, did not show a statistically significant increase in incidence in the CMV-infected group. This differs from findings in other studies, where a significant increased risk of CRS in the CMV-infected group was observed [31-33]. This discrepancy may be due to the limitations of using a database such as NRD, which may not capture detailed clinical information on the type of specific CAR-T product, severity, and management of complications. Notably, after adjusting for complications, encephalopathy was not linked to increased mortality in the CMV-infected group.

American Society of Transplant and Cellular Therapy (ASTCT) recommends CMV screening prior to CAR-T and weekly CMV surveillance in the first two to six weeks post CAR-T in high-risk patients underscoring the growing significance of CMV management in ensuring the success of CAR-T therapy [42]. This study reinforces the critical need for vigilant CMV monitoring and management in CAR-T cell recipients, particularly in those with higher comorbidity burdens. While these findings highlight the potential impact of proactive strategies to prevent CMV reactivation on mortality and prolonged hospitalization, the retrospective nature of the study warrants cautious interpretation, and the results should be further validated in prospective studies.

Limitations

Our study had several limitations. First, the study reinforces the critical need for vigilant CMV monitoring and management in CAR-T cell recipients, particularly in those with hpective observational design may have introduced a selection bias, and causality could not be determined. Second, the NRD does not provide data on laboratory values, administered medications, specific CAR-T therapy types, or treatments for managing complications, which limits the evaluation of their influence on outcomes. Third, the distinction between CMV viremia and disease, along with the treatment of the majority of viremia cases on an outpatient basis, is beyond the scope of this inpatient database study, and ICD coding may not accurately reflect clinical practice. Lastly, the relatively short follow-up period may have failed to capture the long-term incidence or effects of CMV infection.

## Conclusions

In summary, this study underscores the considerable burden of CMV infection in hospitalized CAR-T cell therapy recipients, with a notable association with higher mortality rates and extended hospitalization. CMV infection remained significantly associated with increased mortality and longer length of stay even after accounting for other treatment-related complications. These findings highlight the importance of early detection and targeted management of CMV in CAR-T recipients, particularly those with higher comorbidity burdens, to optimize patient outcomes and minimize the impact of CMV on the overall clinical course. However, given the retrospective design of this analysis, these observations should be interpreted with caution and warrant confirmation in prospective studies.
